# Does historical data still matter for demand forecasting in uncertain and turbulent times? An extension of the additive pickup time series method for SME hotels

**DOI:** 10.1057/s41272-023-00421-1

**Published:** 2023-03-11

**Authors:** Cindy Yoonjoung Heo, Luciano Viverit, Luís Nobre Pereira

**Affiliations:** 1 EHL Hospitality Business School, HES-SO/ University of Applied Sciences and Art Western Switzerland, Lausanne, Switzerland; 2 Hotelnet, Milan, Italy; 3 Research Centre for Tourism, Sustainability and Well-being & Escola Superior de Gestão, Hotelaria e Turismo, Universidade do Algarve, Faro, Portugal

**Keywords:** Hotel demand forecast, Additive pickup, Time series, COVID-19 pandemic, Small and medium-sized enterprises (SMEs) hotels, Revenue management

## Abstract

Demand forecast accuracy is critical for hotels to operate their properties efficiently and profitably. The COVID-19 pandemic is a massive challenge for hotel demand forecasting due to the relevance of historical data. Therefore, the aims of this study are twofold: to present an extension of the additive pickup method using time series and moving averages; and to test the model using the real reservation data of a hotel in Italy during the COVID-19 pandemic. This study shows that historical data are still useful for a SME hotel amid substantial demand uncertainty caused by COVID-19. Empirical results suggest that the proposed method performs better than the classical one, particularly for longer forecasting horizons and for periods when the hotel is not fully occupied.

## Introduction

Forecasting hotel demand has always been challenging, because there are many external factors that can impact hotel demand. In particular, the COVID-19 pandemic has been a huge challenge for the hotel industry, because historical demand data has little value. Industry practitioners argue that even an RM system could not work well because there was no historical data to refer to. Most SME hotels have been struggling to manage the uncertainties of doing business in a world that has been turned on its head by the COVID-19 pandemic. Indeed, it is important that the chosen time series forecasting model can yield accurate forecasts for dates over a medium- and short-term forecasting horizon (1–4 weeks), regardless of the contextual factors.

The forecasting approaches using advanced booking data are typically found in the RM literature and have been applied consistently over the years (e.g., Weatherford and Kimes 2003; Atiya and Gayar 2008; Tse and Poon 2015; Lee 2018; Fiori and Foroni 2019, 2020). Room reservations usually follow similar patterns of the past. Therefore, small hotels tend to forecast occupancy for a given day used to be the occupancy recorded for the same date of the previous year. However, an unprecedented demand environment caused by the pandemic has made the forecasting process even more difficult. Historical data lost some of its value and forecasting has become even more complex, as demands are changing quickly and unpredictably (Kourentzes et al. 2021). Thus, new hotel occupancy forecasting methods should be based not only on historical data but also on current booking status (i.e., reservations on hand).

Several studies have compared the performance of different forecasting models for hotel demand. According to Weatherford and Kimes’ study (2003), pickup methods and regression yielded the lowest error compared to the booking curve and combination forecasts. Ellero and Pellegrini (2014) found that forecasting models based on booking information performed better than historical ones and additive pickup models achieve the best results in the Italian market. In addition, they proposed to investigate possible variants of the pickup models. Following this approach, Fiori and Foroni (2020) extended the multiplicative pickup method based on generalized linear models and tested it with actual booking data from one Italian hotel. As traveler’s booking patterns such as booking window has been dramatically change due to high uncertainty, using only methods strongly dependent on the “same day last year” booking data is not very relevant for hotel forecasting (Webb et al. 2020; Zhang and Lu 2022). While several scholars proposed and tested various demand forecasting models for hotels, no study has explored whether historical data and pickup forecasting models may work during the COVID-19 period. Therefore, this study focused on expanding on the additive pickup time series method for SME hotels and the efficacy of this method was tested using the real reservation data.

## Methodology

### Data

The data used in this research are actual booking data from a small independent hotel on Isola d’Elba, a summer holiday destination in Italy, for six consecutive years (i.e., 2015–2020). The hotel is 3-star property with 32 rooms, open from April to September, which provided booking data.

**Fig. 1:**
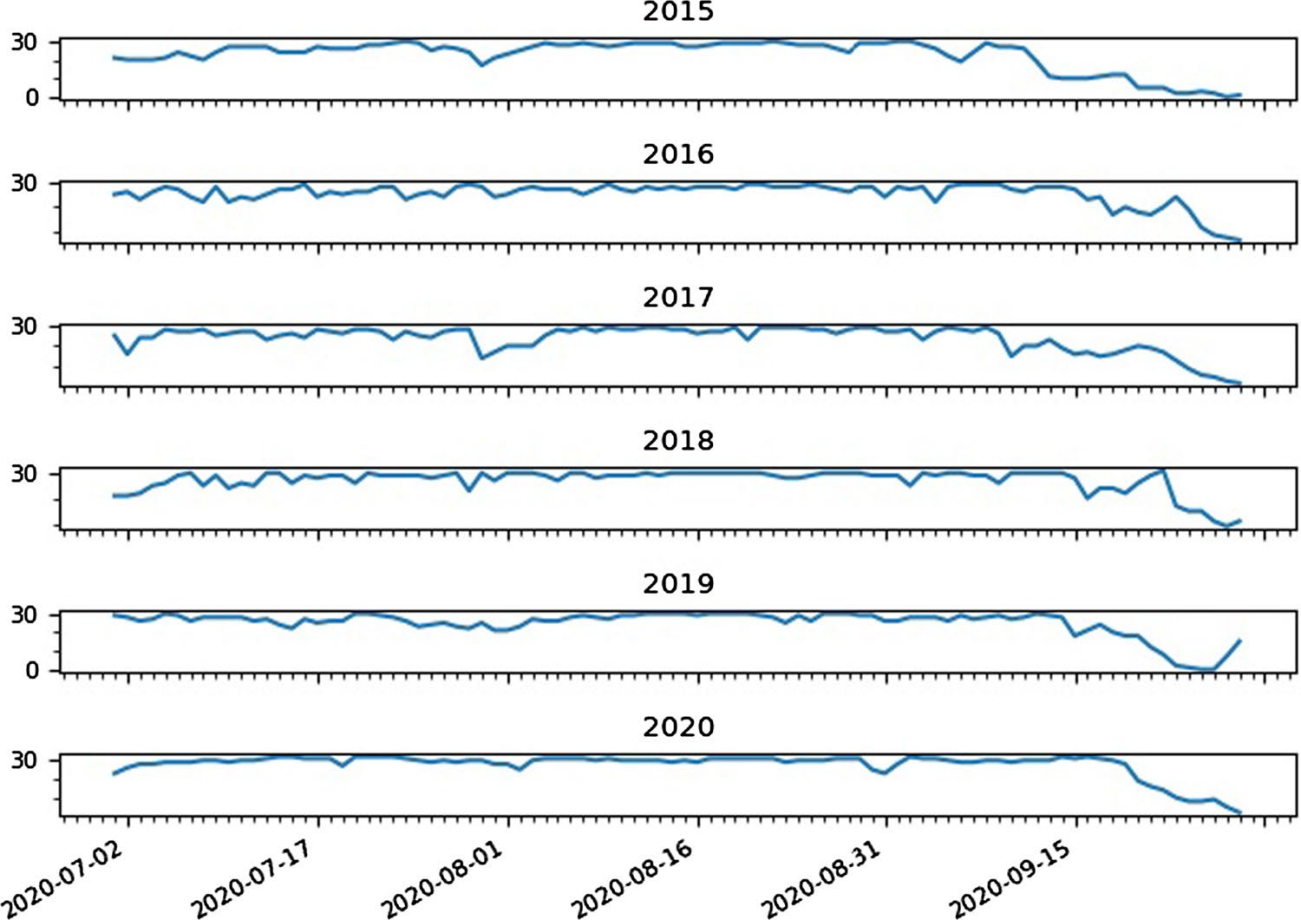
Room occupancy between 2015 and 2020

**Fig. 2:**
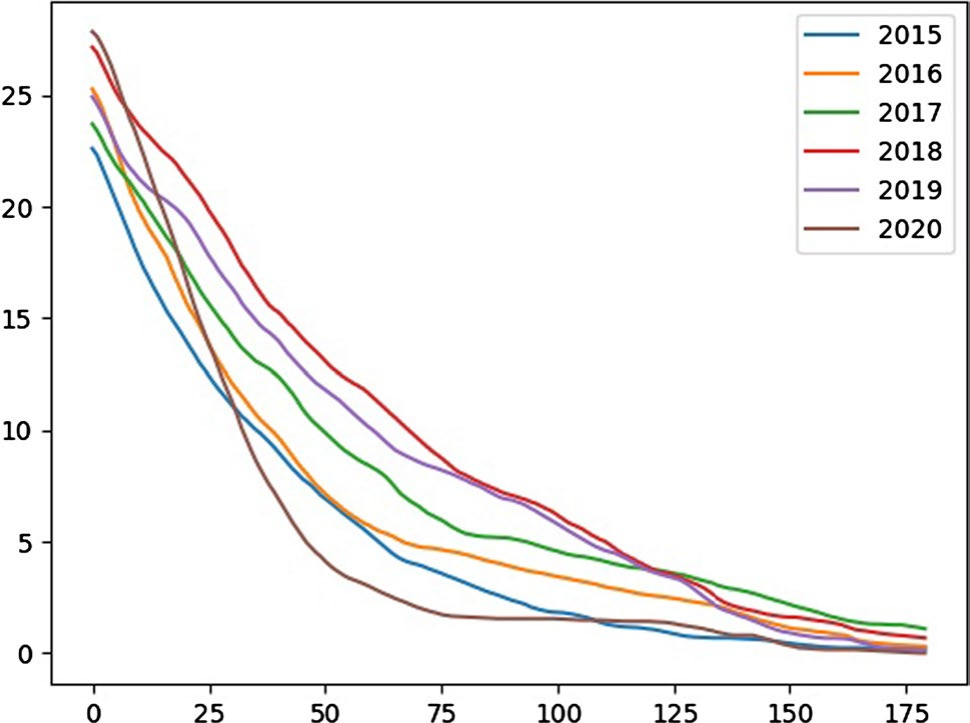
Average booking curves between 2015 and 2020

The time series of daily occupancy data is depicted in Fig. 1, which shows seasonal patterns. We show the annual average booking curve of the hotel per day of the booking window in Fig. 2. Since the high season of the destination is from the beginning of July until mid-September, we have decided to use this period in the empirical experiment. Data from 2015 to 2019 were used to feed the forecasting methods, while data from 1 July to 15 September 2020 were used to evaluate post-sample accuracy of forecasts up to 28 days ahead.

### Methods

The classic additive pickup method predicts a “pickup average” of incremental bookings for each future day that is added to the current bookings on hand for a specific reading day to forecast hotel demand for future dates (Atiya and Gayar 2008; Fiori and Foroni 2019). Daily predictions of incremental bookings to be picked up from a given reading day until a future day of check-in are usually based on a fixed moving average of incremental bookings that have been observed for each specific number of days in advance in the current year (Fiori and Foroni 2019). As a result, forecasts of the number of rooms occupied do not take advantage of the information about the booking path of same calendar period in previous years. Thus, we propose an extension of the classic additive pickup method that predicts daily incremental bookings to be picked up using a moving average of *k*-neighbor periods of the future date of check-in and historical daily records on how bookings behaved in *N* previous years.

Let *b*_*n*,*t*,*j*_ represent the number of rooms booked for the *t*-th check-in day in year *n* (*n* = 0, …, *N*) at least *j* days in advance (*j* = 0, 1, …, *J*). The daily incremental rooms booked between lead times *j* and *j*-1, for *j* = 1, …, *J*, is given by:

$$a_{{n,t,j}} = b_{{n,t,j - 1}} - b_{{n,t,j}}$$

Assuming a moving average of *k*-neighbor periods, incremental bookings between lead times *j* and *j* − 1 in year *n*, for *j* = 1, …, *J* and *n* = 1, …, *N*, is given by:

$$\bar{a}_{{n,t(j)}} = \frac{1}{k}\sum\limits_{{i = t - (k - 1)/2}}^{{t + (k - 1)/2}} {a_{{n,i,j}} }$$

while in the current year (*n* = 0) that quantity is computed by:

$$\bar{a}_{{0,d(j)}} = \frac{1}{k}\sum\limits_{{i = d - (k - 1) + j}}^{{d + j}} {a_{{0,i,j}} }$$

where *k* is a small and odd number of periods in a neighborhood of the future check-in day *t*. With an even number of periods the above moving averages are computed by averaging each pair of uncentered means. We propose that a prediction of incremental bookings between lead times *j* and *j* − 1, for a future check-in day *t* = *d* + *j* (*j* = 1, …, *J*), made on a specific reading day *d* in the current year, and based on historical data from *N* previous years, is obtained from the following expression:

$$\bar{a}_{{d(j)}} = \frac{1}{{N + 1}}\left( {\bar{a}_{{0,d(j)}} + \sum\limits_{{n = 1}}^{N} {\bar{a}_{{n,t(j)}} } } \right)$$

Finally, the *h*-period-ahead forecast (*h* ≥ 1) of the total number of rooms occupied, *Y*, in that future check-in day is given by:

$$\hat{y}_{{d(h)}} = b_{{0,t,h}} + \sum\limits_{{l = 0}}^{{h - 1}} {\bar{a}_{{d(l)}} }$$

where *h* = *t* *−* *d*.

Regarding the evaluation metrics, we resort to the mean absolute percentage error (MAPE), the root mean squared error (RMSE), and the symmetric mean absolute percentage error (SMAPE). The classic additive pickup method is used as a benchmark in the accuracy evaluation.

## Results

Table 1 shows the post-sample forecasting accuracy of the three methods for multi-step forecasts from 1 to 28 days ahead. First, it is important to note that this table reports three accuracy measures revealing similar relative performance of the forecasting methods for all horizons. Results show that the moving average additive pickup method, with both 7 and 14 observations, appear to have the same forecast performance for the whole forecasting horizon. Although the forecasts for one to three days ahead generated by both methods perform similarly, the moving average additive pickup method outperforms the classic additive pickup method for forecasting horizons of at least seven days ahead. Forecasting accuracy gains of the moving average pickup methods are stronger for longer forecasting horizons.

**Table 1:** Summary of accuracy measures of multi-step ahead forecasts for the test sample

Accuracy measure	Forecast horizon	Forecasting method		
		Ad	Ad_MA7	Ad_MA14
RMSE	1 day	0.9	0.9	0.9
	3 days	1.3	1.2	1.2
	7 days	2.5	1.2	1.2
	14 days	5.3	1.2	1.1
	28 days	9.3	1.9	1.9
MAPE	1 day	2.3%	2.1%	2.1%
	3 days	3.3%	3.1%	3.1%
	7 days	5.5%	3.0%	2.9%
	14 days	11.9%	3.1%	3.1%
	28 days	23.9%	4.6%	4.7%
sMAPE	1 day	2.3%	2.1%	2.1%
	3 days	3.3%	3.1%	3.1%
	7 days	5.8%	3.0%	2.9%
	14 days	13.7%	3.1%	3.0%
	28 days	29.9%	4.4%	4.4%

*Ad* classical additive pickup, *Ad_MA7* additive pickup using moving averages of 7 periods, *Ad_MA14* additive pickup using moving averages of 14 periods

Table 2 gives the forecasting accuracy results of the classical and the seven-day moving average pickup methods for multi-step forecasts from 1 to 28 days ahead, per month. Results are consistent with those presented in Table 1, since the moving average method performs better than the classic additive pickup method for longer forecasting horizons. The results also reveal that these accuracy gains are stronger in July and September than in August (which is the peak of high season).

**Table 2:** Accuracy measures of multi-step ahead forecasts by month and forecasting method

Accuracy measure	Forecast horizon	July		August		September	
		Forecasting method		Forecasting method		Forecasting method	
		Ad	Ad_MA7	Ad	Ad_MA7	Ad	Ad_MA7
RMSE	1 day	0.8	0.9	0.7	0.5	1.4	1.4
	3 days	1.2	1.2	1.0	0.9	2.0	1.7
	7 days	2.5	1.3	1.1	1.0	4.1	1.2
	14 days	6.3	1.1	2.3	1.2	7.4	1.0
	28 days	12.6	2.0	3.6	2.1	9.6	1.5
MAPE	1 day	2.1%	2.3%	2.2%	1.5%	3.2%	3.1%
	3 days	2.8%	3.1%	2.9%	2.6%	4.9%	4.2%
	7 days	5.5%	2.9%	3.3%	3.0%	9.9%	3.0%
	14 days	15.2%	2.9%	5.6%	3.7%	18.1%	2.4%
	28 days	37.2%	4.5%	8.3%	5.4%	28.7%	3.3%
sMAPE	1 day	2.1%	2.3%	2.2%	1.5%	3.3%	3.2%
	3 days	2.9%	3.1%	2.9%	2.6%	5.1%	4.3%
	7 days	5.9%	2.9%	3.3%	3.0%	10.9%	3.0%
	14 days	17.8%	2.8%	5.8%	3.6%	21.6%	2.3%
	28 days	48.4%	4.2%	9.1%	5.2%	34.5%	3.2%

## Conclusion

Accurate demand forecasts allow hotels in managing room rates and inventories in a much more effective fashion. The Covid-19 pandemic disrupted tourism and hospitality sectors worldwide through border closures, social distancing measures, the cancelation of transportations, and changes in travelers’ behavior. Indeed, Deyá-Tortella et al. (2022)’s a longitudinal analysis revealed that the COVID-19 pandemic affected hotel guests’ booking patterns such as booking window, distribution channel, and length of stay. Therefore, the demand forecasting models using a traditional approach without accounting for the new conditions caused by exogenous factor like the COVID-19 pandemic cannot estimate the demand correctly.

In this research note, we present an extension of the additive pickup method using moving averages and using historical data. When exogenous factors affect overall tourism demand and hotel booking patterns, historical booking data have a value only when it is properly analyzed. This study shows that historical data are still useful even when there is a high degree of uncertainty because of demand volatility (i.e., COVID-19). Empirical results of our study suggest that the proposed moving average pickup method outperforms the classical one, particularly for longer forecasting horizons and for periods when the hotel is not fully occupied. Thus, we advise SME hotels to use the new moving average pickup method in their forecasting tasks, particularly in periods with strong volatility in hotel demand.

This research note provides important practical implications for SME hotels, which are more threatened by increasing demand uncertainty and global competition than other hotels, and usually cannot afford for a subscription of a full revenue management system. First, this research note suggests a useful instrument for revenue managers maximizing revenue. The additive pickup time series method using moving averages generates accurate forecasts up to 28 days ahead of the date of arrival, and for periods when the hotel is not fully occupied, which is comprehensive and critical information for decision-makers regularly update their operational decisions related to price and inventory allocation. Second, the proposed method requires less data than traditional time-series approaches and can be easily implemented even in MS Excel. There characteristics are particularly pertinent for SME hotels, since they can empower their revenue management decisions with low resources. Finally, the extension of the pickup method is attractive for implementing by programmers of revenue management systems, since the formulae are straightforward, and the method is not computer-intensive.

From a theoretical point of view, the proposed method closes the gap of classical pickup methods of not considering in the forecast incremental bookings that were observed for the same calendar period, in each specific number of days in advance, from previous years. Thus, the main contribution of this research note to the theory of revenue management relies on the use of historical information about the booking path of same calendar period in the additive pickup method, in order to improve accuracy of daily demand forecasts.

Several limitations of the present research should also be noted. This study only applied the additive pickup using moving averages of 7 and 14 periods. The optimal periods of moving average and the moving average pickup method we proposed here may not work for some hotels which target different markets (e.g., business hotels in the major city). Also, the impact of COVID-19 on hotel demand was not shared equally around the world. The hotel we investigated in this study located in a summer holiday destination and enjoyed high occupancy during the summer of 2020 although the booking pattern changed significantly. However, many hotels in the major cities targeting mainly business segments had struggled during the summer of 2020. Further, the impacts of other exogenous factors on overall tourism demand and hotel booking patterns can be different from COVID 19. Therefore, we encourage future research to apply the proposed approach to various contexts and improve it further. For example, future research may consider applying the new additive pickup method per cluster of booking curves. Pattern similarity-based machine learning methods can be used to cluster booking curves. It would be interesting to compare hotel demand forecasting accuracy of this method with a cluster-based method that uses data of only similar stay days (i.e., belonging to the same cluster) to generate forecasts.
